# A Survey of Parents’ Perspective of Receiving a Developmental Diagnosis for Their Child

**DOI:** 10.3390/children12010105

**Published:** 2025-01-17

**Authors:** Stacey D. Miller, Maria Juricic, Jaimy Coates, Jeffrey N. Bone, Ash Sandhu, Kishore Mulpuri, Maureen O’Donnell

**Affiliations:** 1 BC Children’s Hospital, Vancouver, BC V6H 3V4, Canada; 2 Department of Physical Therapy, University of British Columbia, Vancouver, BC V6T 1Z3, Canada; 3 BC Children’s Hospital Research Institute, Vancouver, BC V5Z 4H4, Canada; 4 Department of Orthopaedics, University of British Columbia, Vancouver, BC V5Z 1M9, Canada; 5 Department of Orthopaedic Surgery, BC Children’s Hospital, Vancouver, BC V6H 3N1, Canada; 6 Biostatistics, Clinical Research Support Unit, BC Children’s Hospital Research Institute, Vancouver, BC V5Z 4H4, Canada; 7 Sunny Hill Health Centre for Children, Vancouver, BC V6H 3N1, Canada; 8 Department of Pediatrics, University of British Columbia, Vancouver, BC V6H 3V4, Canada

**Keywords:** diagnosis, cerebral palsy, caregiver experience

## Abstract

Receiving a diagnosis, such as cerebral palsy (CP), can have lasting impacts on caregivers and families. Previous literature has described that caregivers wish to receive a diagnosis together, without delay, in a private, direct, honest, and sympathetic way. This study aimed to understand the experience of caregivers of children with cerebral palsy (CP) or similar conditions when receiving a diagnosis for their child.: Caregivers of children and youth aged 0–18 years with CP or a similar physical condition completed a survey investigating who first provided a diagnosis, and the way it was provided. Questions were based on the pediatric SPIKES framework. Caregivers were asked how receiving a diagnosis could have been improved. Thematic analysis of qualitative data were performed.: A total of 180 surveys were completed. Mean age at diagnosis was 19.0 (SD 25.7) months. Most caregivers reported the healthcare provider was sympathetic and understanding (75%) and clear and direct (69%). Only 25% of caregivers recalled being directed to where to find information; only 37% reported being told what their child does well or is expected to do well. Thematic analysis revealed three themes: (1) less waiting, (2) more compassionate communication, and, (3) information and support to move forward: Caregivers wanted the diagnosis to be timely and provided with compassion. Requests for greater information on the diagnosis, available support, and their child’s future, suggest that greater care to support families in the aftermath of receiving a diagnosis is also needed.

## 1. Introduction

Sharing information with parents about their child’s life-long diagnosis can be a challenging and momentous task. While families report that having a diagnosis is beneficial, the process of receiving the diagnosis can be distressing [1,2]. When sharing “bad news” is conducted poorly, there can be lasting emotional injury for parents [3,4]. Parents may experience depression, confusion, long lasting distress, and resentment if communication of bad news is not conducted well [3,5]. Parents’ emotions, beliefs, and attitudes towards medical staff, the parent–child relationship, and how well a parent resolves the diagnosis can be influenced by how a diagnosis is provided [5].

Parental dissatisfaction with how a diagnosis is disclosed is preventable [6]. Previous findings have identified that parents wish to receive a diagnosis together, without delay, in a private, direct, honest, and sympathetic way [6,7,8,9]. Pediatric specific guidelines that describe how to approach and structure conversations that involve sharing life altering information have been developed. Based on the SPIKES protocol, originally designed for use by oncologists sharing diagnostic and prognostic information with adult patients [10,11], the pediatric guidelines described by Wolfe et al., 2014 provides 33 recommendations across six components: S—Setting, P—Perception, I- Involvement, K—Knowledge, E—Emotion, empathy, and S—Strategy, summary, self-reflection [11]. The adaptations made to the SPIKES protocol to create the guidelines for use in a pediatric setting emphasize a patient- and family-centered approach that is culturally sensitive to each family. Families are encouraged to ask questions, and the care provider should emphasize shared goals and facilitate discussion amongst those involved [11]. 

The objective of this study was to understand the experience of caregivers when receiving a diagnosis of cerebral palsy (CP) or another similar physical disability for their child. We aimed to learn the age at which children had been diagnosed, and the way a diagnosis was provided utilizing criteria described in the pediatric SPIKES guidelines. We also sought to learn how the process could have been made easier for families.

## 2. Materials and Methods

The study was reviewed and approved by the local Research Ethics Board. No research funding was received for this study. Participants were caregivers of children and youth aged 0–18 years old with CP or another similar physical disability. Utilizing consecutive sampling, all caregivers attending an Orthopaedic clinic at a Canadian tertiary care pediatric hospital of children that met the inclusion criteria were invited to participate between December 2019 and September 2020. Caregivers who did not have adequate English language skills used an interpreter. Those consenting to participate completed the survey via tablet computer. Additionally, parents and caregivers enrolled in the local hip surveillance program who indicated interest in research were mailed or emailed, depending upon their preference, an invitation to participate with the survey link. 

Participants were first asked to identify, from a list of seven childhood disorders, which diagnosis best described their child’s medical diagnosis. Alternatively, participants could select “other” and enter a diagnosis or select that they had never been given a diagnosis for their child. Caregivers were also asked if their child had been provided additional diagnoses. For those who provided a diagnosis, questions first addressed age at diagnosis, presenting symptoms, concerns about development prior to diagnosis, and services received prior to diagnosis. Subsequent survey questions were developed using the pediatric guidelines for sharing life-altering information, and explored who first provided a diagnosis and the manner in which it was provided [11]. Multiple choice, yes or no, and Likert scale (5-point) questions were utilized. The survey concluded with open-ended questions regarding how the experience could have been made better for caregivers. If participants reported their child did not have a diagnosis, caregivers were asked what concerns they had about their child’s health and development and the age when they first had a concern. Child and caregiver demographics were collected from all respondents. 

Data collected in the survey were captured and stored electronically via a REDCap electronic data capture tool [12]. Descriptive statistics were used to summarize the cohort overall, and by diagnosis. Means and standard deviations were used for continuous variables and counts and frequencies for categorical variables. To assess potential differences in location and time on the experience of receiving a diagnosis, a proportional odds model was fit to the agreement (Likert) responses for those diagnosed (1) within and outside of the province and (2) in the past 5 years verses greater than 5 years ago. We verified the proportional odds assumption, and the results are presented as odds ratios and 95% confidence intervals.

A thematic analysis of qualitative data were performed [13]. The comments were first read and coded using recurrent issues in the text. When one response consisted of more than one issue, the comment was separated and coded into multiple comments. Abstract themes were then established, followed by theme refinement. Coding was performed by one author (JC) and then audited by a second author (MJ). When the second reviewer interpreted the comments differently, a dialog occurred between the reviewers to refine the coding and themes. Discussion amongst multiple authors (JC, MJ, and SDM) was utilized to finalize the organizing and global themes. A thematic network was then constructed.

## 3. Results

In total, 175 families were approached in the clinic with 116 agreeing to participate. Invitations to participate were emailed to 237 caregivers and mailed to 223 caregivers of children enrolled in the provincial hip surveillance program for children with CP of which 64 surveys were completed. In total, 180 caregivers participated in the survey for an estimated overall response rate of 28%. Twelve surveys were incomplete; available data from these surveys were utilized.

### 3.1. Quantitative Data

Characteristics of children and survey respondents are shown in Table 1. Thirty-six (20%) respondents reported an “other” diagnosis for their child. More than half of these were etiological diagnoses consistent with the definition of CP; four of these caregivers reported a secondary diagnosis of CP. Overall, the mean age at diagnosis was 19.0 months (SD 25.7).

The diagnosis was provided in the local province for 144 (84%) children; 26 (15%) caregivers reported receiving the diagnosis elsewhere. Children diagnosed locally were, on average, older at diagnosis at 20.0 months (SD 26.7) compared to 13.2 months (SD 18.2) (*p* = 0.004). The first language was also significantly different (*p* < 0.001), with English being the first language for 44% of respondents whose child was diagnosed outside the province, verses 80% for those diagnosed in the province. Of those reporting they had received a diagnosis for their child, 62 caregivers (36%) reported the diagnosis was provided within the last 5 years while 97 (57%) reported receiving the diagnosis more than 5 years ago; the time since diagnosis could not be determined for 21 (12%) participants (Table 2). Children diagnosed within the last 5 years were less likely to have received a diagnosis of CP, fewer were diagnosed by pediatricians, and the mean age at diagnosis was older (Table 2).

Questions related to caregiver satisfaction with the process of receiving a diagnosis were similar based on location (OR = 0.89, 95% CI = [0.42, 1.87], *p* = 0.76) and time since diagnosis (OR = 0.94, 95% CI = [0.45,1.98]; *p* = 0.51), so data are presented together (Table 3 and Table 4). 

### 3.2. Qualitative Comment

**Figure 1:**
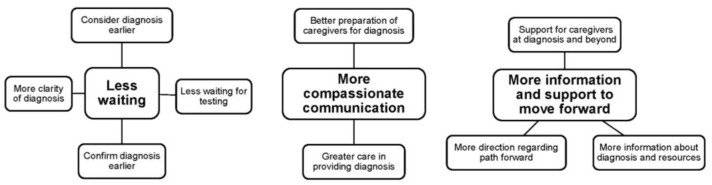
Thematic network diagram of thematic analysis for responses to the question: “What, if anything, could have been done to make getting the diagnosis easier?”. Organizing themes are shown branching from three global themes.

Open-ended questions were completed by 107 participants. Responses were divided into 197 comments answering the question, “what, if anything, could have been done to make getting the diagnosis easier?”. Upon completing the thematic analysis, three themes were revealed: (1) less waiting, (2) more compassionate communication, and (3) information and support to move forward. The thematic network diagram with the overarching question, three global themes, and organizing themes is shown in Figure 1.

#### 3.2.1. Less Waiting

A recurring theme of frustration emerged from caregiver comments pertaining to delays in various aspects of care. There was delay in acknowledging parental concerns, obtaining diagnostic testing, including genetic testing and medical imaging, and confirming a diagnosis. Caregivers expressed their concerns were not taken seriously with one caregiver noting they “complained for a long time before anyone listened”. The “wait and see” approach to diagnosis was distressing and prolonged the uncomfortable feeling of the unknown. One participant noted “I would have preferred to have a tentative diagnosis of what it appears to be, so I could have been dealing. Rather, I was left apprehensive and concerned about all the problems we were having, trying to ‘catch up’, and getting more and more concerned about increasing delay”. The lack of information had the potential to mislead families as described by one parent who stated: “no one confirm[ed] with us yet that our son had CP. Before the diagnosis of CP, we had an impression that our son’s condition of low/high tone would be improving close to 100%”.

Some caregivers reported knowing their child’s diagnosis before it was given by a physician while others had to ask for confirmation of a diagnosis. For some, providers were either vague or provided “a vast range of possibilities”. Caregivers wished confirmation could have been given earlier and with more clarity as illustrated by a participant’s comment that “diagnosis itself was entirely expected for us. By the time we were able to sit in the office with the Neurologist and receive the official diagnosis I had already “known” for a year. Down to the exact subtype of Cerebral Palsy. In fact, I told the doctor what I thought it was and she confirmed immediately”.

#### 3.2.2. More Compassionate Communication

Seeking more compassionate communication when interacting with providers was the second global theme. It was distressing for caregivers when informed of their child’s diagnosis for the first time in a casual way, such as over the phone, in a letter, while all parties were standing, or in a busy environment. Caregivers identified the importance of being prepared for receiving the diagnosis. One caregiver noted that “when given his diagnosis, I hadn’t even completely entered the room, I wasn’t even sitting down yet when she told my husband and I. It would have been nice if I was first sitting” while another described how the provider “just said it and we were shocked”.

For some, it was assumed that they knew the diagnosis, or the term CP was used without the family being given the diagnosis. One participant noted “it was as though the doctor expected us to already know. The information was casually written on his form as if it was just understood, just a known fact about my child”. Another participant described that “many specialists had used the term of Cerebral Palsy when [her daughter] visited them, and I asked why they were stating that on her chart? I didn’t know what it was at the time. So everyone knew prior to us actually receiving an official diagnosis”.

For some caregivers, the manner in which the diagnosis was provided lacked hope and empathy. A participant explained that they “found that all the appropriate people were there, but the diagnosis was provided in a ‘grim’ and ‘doom & gloom’ way...I’m sure there is no perfect way to deliver information of this magnitude and I’m sure they don’t want to give false hope, but sometimes hope is what families need. Our son is severely affected, and I still think he deserves hope and positivity and more focus on what he CAN do. Possibly more of a positive focus would be helpful”. Another participant noted that “because of the rarity of our son’s particular diagnosis the geneticist almost seemed excited to tell us of his genetic syndrome which really bothered me”.

#### 3.2.3. Information and Support to Move Forward

Caregivers wished for more information regarding their child’s diagnosis and what could be done to support their child. They wanted to educate themselves but felt that they lacked resources. In particular, information, including written materials about the diagnosis, therapy services, family support, and funding were highlighted. Respondents shared that they did not know what to expect for their child’s future with one participant stating “It would have also been nice to have been given a folder of some sort of contact info for support services and maybe support groups for families of CP so we could connect and hear other experiences and meet other children with varying degrees of CP”.

Support for parents was identified as a gap. This included having a support person available when receiving the diagnosis. One respondent noted “we were handed a life-changing diagnosis, while going over brain scans that were awful to see, and then she just left”. Additionally, follow-up after having “the opportunity to process the diagnosis” was seen as desirable.

## 4. Discussion

The guidelines developed for pediatric settings based on the SPIKES protocol were used to learn about the manner in which families had received a diagnosis for their child. Most caregivers agreed that the diagnosis was given in a clear and direct way and that healthcare providers encouraged questions. While most caregivers were satisfied with the “Setting” in which the diagnosis was provided and with those present, caregivers in our study identified that they were not always prepared to receive a diagnosis for their child. Caregivers reported that professionals used a diagnosis verbally or in written communications prior to the diagnosis being provided to them. As described in the pediatric SPIKES guidelines, the “Perception” and “Involvement” components encourage providers to first explore what the family already knows about a child’s medical condition, tailor information to the family, and provide a “warning shot” [11]. Baile et al., 2000 describe this as implementing the axiom “before you tell, ask” [10]. It is incumbent on all members of a child’s healthcare team to assess caregivers’ understanding of a child’s diagnosis at their first meeting. Dagenais et al., 2006 found parental satisfaction with the process of communicating a diagnosis in children with CP was related to the quantity and content of information given [7]. Respondents identified that they wanted more information or “Knowledge”. Most caregivers reported that the provider ensured they understood the information being shared and did not use medical terms. As in other studies, caregivers wanted providers to be caring and offer hope, consistent with the “Emotion, Empathy” components of the guidelines [11,14,15]. Although most caregivers felt the healthcare provider was sympathetic and understanding, the global theme of “more compassionate communication” from the thematic analysis indicates that providers’ communication did not always convey this and can be improved. 

The second “S” within the SPIKES protocol relates to strategy, summary, and self-reflection [11]. The aim is to offer options to the family and, together, create a plan for moving forward. Caregivers reported this was lacking. Only 25% of respondents recalled being directed to where to find information and less than half recalled having a follow up appointment scheduled. Having “information and support to move forward” was one of the global themes identified from caregiver comments and, when asked what they were not provided but wished to receive, the most common responses were internet resources (57%), available family supports (49%), and written information (47%). The request for written information and support has been reported in previous studies and should be given greater priority by clinicians [4,14,16].

When asked what could have been done to make getting the diagnosis easier, the desire for less waiting was identified as a global theme. Greater rates of parental depression, stress, and overall dissatisfaction with diagnosis have been found when delivery of the diagnosis is delayed [4]. A diagnosis may allow caregivers to regain control and develop active coping strategies and benefit parental mental health [17]. Standardized tools are now available to allow early and accurate diagnosis of CP. Neonatal magnetic resonance imaging (MRI), the Prechtl Qualitative Assessment of General Movements (GMs) and the Hammersmith Infant Neurological Examination (HINE), have high predictive validity for risk of CP while MRI and the HINE are most predictive of risk for cerebral palsy after 5 months’ corrected age [18]. Novak et al. [18] recommend that an experienced clinical team conduct and interpret the standardized assessments and communicate the results. In our province, a new interdisciplinary clinic has been established for high-risk infants, under the age of one, with developmental pediatricians, a nurse clinician, a physiotherapist, an occupational therapist, and a social worker to meet this need. Some children, such as those with mild motor impairments or those not requiring extended hospitalization after birth, tend to be referred for diagnosis much later [19]. An international expert panel has identified clinical features that should prompt referral, “warning signs” that require monitoring, and referral recommendations to other professionals that should occur at the time of referral for diagnosis [20]. These recommendations provide direction for primary care physicians in identifying who requires diagnostic assessment and support parents in our study who reported feeling their concerns were not acknowledged.

Interestingly, children who had received a diagnosis within the 5 years prior to completing the survey were less likely to receive a diagnosis of CP and were older at diagnosis. More children were provided a diagnosis by a geneticist and fewer were provided a diagnosis from a general pediatrician. These findings suggest that the search for an etiological diagnosis and referral to sub-specialists for a diagnosis is common and may lead to increased length of time until diagnosis. Additionally, it illustrates that there is increased focus on the genetic causes of CP [21]. Importantly, caregivers in our study expressed that they want clarity as to why their child has CP. Unfortunately, in many cases, etiology remains unknown and delaying a clinical diagnosis of CP until investigations are complete may increase parental distress and limit access to condition specific early interventions and supports [22]. At the time of diagnosis, 18% of our survey respondents reported their children were not receiving any therapy support services. When a diagnosis of CP cannot be made with certainty, an interim diagnosis of “high risk of CP” may be given to the child to allow access to services earlier at a time when neuroplasticity offers greater opportunity for gains in function and reduce caregiver stress [18,23]. 

Opportunities for future research on parental experience in receiving a diagnosis remain. Future studies should focus on prospectively collected data to evaluate the experience of parents using either mixed-methods or qualitative design to further explore the wishes of parents. Respondents wanted to wait less for a diagnosis but also wanted to understand the etiology of their child’s condition. Parental needs during this period of investigation and acceptance of a diagnosis of “high risk of CP” require further study, including their trust of providers when this diagnosis is removed, or a progressive condition is identified. While most respondents in our survey were caregivers of children with CP or a condition consistent with the definition of CP, there were a small number of caregivers of children with progressive conditions. While the use of the SPIKES framework to deliver the diagnosis would be applicable in both circumstances, it is possible that caregivers of children with a progressive condition may react differently to the information provided and wish for different supports. Additionally, future work should explore whether a child’s level of impairment has an impact on the experience of parents when receiving a diagnosis. Importantly, parent partners should be involved in the design and execution of research in this area.

Overall, caregivers of children with CP and similar neuromotor conditions identified that an early diagnosis, given in a compassionate way, with information and support to allow them to move forward is important when being provided a diagnosis for their child. One participant stated, “we strongly encourage additional education/training for those who share diagnoses with families”. The SPIKES protocol offers concrete methods to guide discussions with families about diagnosis and can be used to educate healthcare providers on how best to approach these important conversations. Table 5 lists recommendations described by Wolfe et al. [11] for sharing life-altering news in pediatric settings and includes quotes from study participants illustrating their importance to caregivers. Integration of these pediatric specific guidelines into medical education and pediatric residency and fellowship training has the potential to have a lasting impact on children and families.

This study has limitations. Respondents were asked to retrospectively report their experience with receiving a diagnosis for their child. For some caregivers, this was many years in the past and could result in recall bias. While almost 60% of respondents provided comments, open-ended survey questions were optional and so qualitative data responses were not collected from all participants. Respondents were from a single Canadian province, which may limit the generalizability of findings. However, responses related to caregiver satisfaction when receiving a diagnosis did not significantly differ for those receiving a diagnosis inside or outside the province, thus, suggesting they are representative of parent experience outside of the local healthcare setting. While the response rate was high for recruitment in person, it was low amongst those approached via mail or email. This may reflect the difference in recruitment methods but, given the overall response rate was low, this further limits generalizability. The low response rate may lead to selection bias. It is unknown if the recruitment method impacted the reported experience of caregivers. We hypothesize that those who found the experience traumatic, and potentially less satisfactory, may be most likely to decline to participate regardless of recruitment method. Despite these limitations, the study’s findings offer insight into the experiences of parents and are important for all clinicians who provide developmental diagnoses.

## Figures and Tables

**Table 1:** Child and caregiver participant demographics.

	Responses *n* (%)
Sex of Child	
Male	97 (53.9)
Female	71 (39.4)
Not reported	12 (6.7)
Medical diagnosis	
Cerebral Palsy	128 (71.1)
Autism	5 (2.8)
Muscular Dystrophy	1 (0.6)
Rett Syndrome	1 (0.6)
Other	36 (20.0)
Never given a diagnosis	9 (5.0)
Child’s current age (years) Mean (SD), range	8.9 (4.3), 0.67–18.0
Current Ambulatory status	
Ambulatory without aid	93 (51.7)
Ambulatory with aid	21 (11.7)
Non-ambulatory	53 (29.4)
Unknown	13 (7.2)
Term delivery	102 (56.7)
Stayed in the NICU	97 (58.1)
Caregiver had concern before diagnosis	134 (74.4)
Gross motor delay	103 (57.2)
Fine motor delay	83 (46.1)
Problems with speech/communication	61 (33.9)
Feeding difficulty	51 (28.3)
Problem with vision	28 (15.6)
Challenging behaviour	23 (12.8)
Problem with hearing	15 (8.3)
Other	20 (11.1)
Respondent	
Biological Mother	126 (70.0)
Biological Father	37 (20.6)
Other/unknown	17 (9.4)
English is first language	
Yes	126 (70.0)
No	42 (23.3)
Unknown	12 (6.7)
Marital status (%)	
Married/Domestic partnership	134 (74.4)
Separated or divorced	17 (9.4)
Single	16 (8.9)
Unknown	13 (7.2)
Highest level of education	
Bachelors	46 (27.5)
Graduate degree	31 (18.6)
Trade school or college diploma	59 (35.3)
High school	24 (14.4)
Did not complete high school	2 (1.2)
Would rather not say	5 (3.0)
Unknown	13 (7.2)
Population of Home Community	
Metro (over 190,001)	86 (47.8)
Urban/rural (40,001–190,000)	50 (27.8)
Rural (10,001–40,000)	21 (11.7)
Remote (0–10,000)	11 (6.1)
Unknown	12 (6.7)

**Table 2:** Significant results when diagnosis within or beyond the last 5 years.

	N	Diagnosis ≤5 Years *n* = 62	Diagnosis >5 Years *n* = 97	Unknown *n* = 21	*p*-Value^2^
Best description of child’s medical diagnosis: *n*, (%)	180				<0.001
CP		39 (63%)	81 (84%)	8 (38%)	
Autism		3 (5%)	2 (2%)	0 (0%)	
Muscular Dystrophy		0 (0%)	0 (0%)	1 (5%)	
Rett Syndrome		0 (0%)	1 (1%)	0 (0%)	
Never been given a diagnosis		0 (0%)	0 (0%)	9 (43%)	
Other		20 (32%)	13 (13%)	3 (14%)	
Age (in months) when child CP diagnosed (median, IQR)	126	18 (6, 24)	10 (2, 18)	1 (1, 10)	0.008
For other diagnoses, age (in months) when diagnosis given (median, IQR)	42	35 (15, 54)	11 (5, 23)	7 (4, 13)	0.061
First provided diagnosis: *n* (%)	170				0.024
Neurologist		20 (32%)	26 (27%)	2 (10%)	
Geneticist		9 (15%)	3 (3.1%)	1 (5%)	
Pediatrician		8 (13%)	30 (31%)	2 (10%)	
Developmental pediatrician		4 (7%)	1 (1.0%)	0 (0%)	
Orthopaedic surgeon		5 (8%)	5 (5.2%)	0 (0%)	
Physical therapist		2 (3%)	3 (3.1%)	0 (0%)	
Family physician		0 (0%)	5 (5.2%)	1 (5%)	
Occupational therapist		0 (0%)	1 (1.0%)	0 (0%)	
Other		8 (13%)	10 (10%)	4 (19%)	
I don’t remember		6 (10%)	13 (13%)	1 (5%)	
Unknown				10 (48%)	

**Table 3:** Responses related to the manner in which diagnosis was provided.

Survey Question	All Diagnoses *n* = 171
Diagnosis was given at first visit with provider	53 (31%)
Who else was present?	
Second parent	88 (52%)
Your child	66 (39%)
Another doctor	21 (12%)
Grandparent(s)	18 (11%)
Student doctor	14 (8%)
PT	13 (8%)
Other	11 (6%)
No one else	30 (18%)
Believed right people were present at time of diagnosis	149 (87%)
Location when diagnosis given:	
Doctor’s office	50 (29%)
Hospital clinic examination room	47 (28%)
Family/meeting room at hospital	19 (11%)
Hospital inpatient room	13 (8%)
Child development center	11 (6%)
Home	8 (5%)
Other	11 (6%)
I don’t remember	11 (6%)
Blank	1 (0.6%)
Felt location was appropriate	151 (88%)
Healthcare provider was seated	
Yes	101 (59%)
I don’t remember	36 (21%)
Information provided at time of diagnosis	
What the condition is	133 (78%)
Why your child has the condition	83 (49%)
What can be done to support your child (e.g., therapies, medications, or surgery)	79 (46%)
What to expect for your child’s future	72 (42%)
Written information on your child’s condition	39 (23%)
What family supports are available to you	38 (22%)
Web sites/internet resources for you to access	24 (14%)
Other	11 (6%)
Information not provided at time of diagnosis but that wanted	
Web sites/internet resources for you to access	98 (58%)
What family supports are available to you	89 (52%)
Written information on your child’s condition	85 (50%)
What to expect for your child’s future	73 (43%)
What can be done to support your child (e.g., therapies, medications, or surgery)	58 (34%)
Why your child has the condition	44 (26%)
What the condition is	23 (14%)
Were you provided with where to find additional information?	
Yes	42 (25%)
I don’t remember	56 (33%)
Did the healthcare provider offer to help share the information with others (such as siblings, family members, etc.)?	
Yes	24 (14%)
I don’t remember	36 (21%)
Was the follow-up appointment scheduled to review the diagnosis?	
Yes	77 (45%)
I don’t remember	27 (16%)

**Table 4:** Caregivers’ satisfaction with process of receiving diagnosis for their child (*n* = 171).

	Strongly Agree/Agree	Undecided	Strongly Disagree/Disagree
The diagnosis was given in a clear and direct way.	118 (69%)	23 (14%)	29 (17%)
The healthcare provider used medical terms that I found hard to understand.	37 (22%)	36 (21%)	96 (57%)
I had enough time to discuss the diagnosis with the healthcare provider.	94 (55%)	36 (21%)	40 (24%)
The healthcare provider ensured that I understood the information that was shared.	107 (64%)	32 (19%)	29 (17%)
The healthcare provider discussed what my child does well or is expected to do well.	63 (37%)	48 (28%)	58 (34%)
The healthcare provider encouraged me to ask questions.	99 (59%)	36 (21%)	33 (20%)
I felt that the healthcare provider was sympathetic and understanding.	126 (75%)	23 (14%)	19 (11%)
I felt that the healthcare provider was hopeful.	98 (58%)	48 (28%)	23 (14%)
I felt that the healthcare provider was sensitive to my culture and my religious beliefs.	94 (56%)	61 (37%)	12 (7%)

**Table 5:** Recommendations for sharing life-altering news in pediatrics and supporting participant quotes.

Domains	Summary of Pediatric Guidelines for Sharing Life-Altering Information [11]	Associated Quotes from Caregivers When Asked: “What Could Have Been Done to Make Getting the Diagnosis Easier?”
S: Setting	Identify who (child, family, healthcare team members, interpreter) will be present; arrange for child life or other caregiver, if needed Be seated in private room, introduce team and roles, avoid interruptions	“It would have been easier if the diagnosis was given in person” “I would definitely have brought someone with me instead of being alone with my child” “Perhaps not a full room of [doctors] in the [Emergency Room] exam room”
P: Perception	Explore what family knows already Correct misinformation Tailor information to family’s level of understanding	“When I was told about the diagnosis I had no knowledge about CP” “My own research before meeting with the doctor already had me prepared”
I: Involvement	Confirm agenda Encourage questions Be sensitive to culture, race, religious beliefs, socioeconomic background	“Not making us feel stupid for questions we had” The experience was very dismissive. No room for questions, no supports, just sent on our way”.
K: Knowledge	Provide warning first Share information about diagnosis and connect with known information Pace information Check understanding periodically Avoid medical jargon Use handouts Highlight positive findings, when able Discuss potential outcomes, including uncertainty in the future	“I had no idea we would be getting a diagnosis when we did” “This completely blindsided me”. “More information on the diagnosis and how it might affect her different body systems” “Paperwork that clearly stated findings would be helpful” “Maybe given some positives... I was told my son would never do anything”
E: Emotion, empathy	Respond empathically (ex. “I can see how upsetting this is for you”.) Clarify thoughts/feeling with exploratory questions (ex. “Could you tell me what you’re worried about?”) Validate emotions (ex. “I can understand how you feel that way”.)	“It was provided in probably the most uncaring disrespectful way”. “She actually asked me, ‘Why are you crying?’” “When the diagnosis was given to me I was upset and scared but my reaction seemed inappropriate gauging by the doctor’s reaction”.
S: Strategy, Summary, Self-Reflection	Determine if caregivers are ready to discuss next steps Offer treatment recommendations Confirm understanding Balance hope and realism Summarize goals, timelines, next steps Offer help sharing diagnosis with others Provide information about support services Follow-up with caregivers Reflect as a team	“My only wish would have been to receive more information on treatment and support” “Offer me hope” “Provide clearer next steps and follow up on diagnosis” “I would like to have had a booked follow up for me and my child’s big sisters” “Social workers and counseling should be a priority”

## Data Availability

The data that support the findings of this study are available from the corresponding author, S.D.M., upon reasonable request.
